# Population distribution and ancestry of the cancer protective MDM2 SNP285 (rs117039649)

**DOI:** 10.18632/oncotarget.1910

**Published:** 2014-04-18

**Authors:** Stian Knappskog, Liv B. Gansmo, Khadizha Dibirova, Andres Metspalu, Cezary Cybulski, Paolo Peterlongo, Lauri Aaltonen, Lars Vatten, Pål Romundstad, Kristian Hveem, Peter Devilee, Gareth D. Evans, Dongxin Lin, Guy Van Camp, Vangelis G. Manolopoulos, Ana Osorio, Lili Milani, Tayfun Ozcelik, Pierre Zalloua, Francis Mouzaya, Elena Bliznetz, Elena Balanovska, Elvira Pocheshkova, Vaidutis Kučinskas, Lubov Atramentova, Pagbajabyn Nymadawa, Konstantin Titov, Maria Lavryashina, Yuldash Yusupov, Natalia Bogdanova, Sergey Koshel, Jorge Zamora, David C. Wedge, Deborah Charlesworth, Thilo Dörk, Oleg Balanovsky, Per E. Lønning

**Affiliations:** ^1^ Section of Oncology, Department of Clinical Science, University of Bergen, 5020 Bergen, Norway; ^2^ Department of Oncology, Haukeland University Hospital, 5021 Bergen, Norway; ^3^ Research Centre for Medical Genetics, Russian Academy of Medical Sciences, 115478 Moscow, Russia; ^4^ Estonian Genome Center, University of Tartu, Tartu, Estonia; ^5^ Department of Genetics and Pathology, International Hereditary Cancer Center, Pomeranian Medical University, Szczecin, Poland; ^6^ IFOM, Fondazione Istituto FIRC di Oncologia Molecolare, Milan, Italy; ^7^ Unit of Molecular Bases of Genetic Risk and Genetic Testing, Department of Preventive and Predictive Medicine, Fondazione IRCCS Istituto Nazionale dei Tumori, Milan, Italy; ^8^ Department of Medical Genetics, University of Helsinki, Helsinki 00014, Finland; ^9^ Department of Public Health, Faculty of Medicine, Norwegian University of Science and Technology, Trondheim, Norway; ^10^ Department of Human Genetics, Leiden University Medical Center, RC Leiden 2300, The Netherlands; ^11^ Department of Pathology, Leiden University Medical Center, RC Leiden 2300, The Netherlands; ^12^ Genetic Medicine, MAHSC, University of Manchester, St. Mary's Hospital, Manchester, M13 OJH, UK; ^13^ Department of Etiology and Carcinogenesis, Cancer Institute and Hospital, Chinese Academy of Medical Sciences and Peking Union Medical College, Beijing 100730, China; ^14^ Department of Medical Genetics, University of Antwerp, Antwerp, Belgium; ^15^ Laboratory of Pharmacology, Democritus University of Thrace Medical School, Alexandroupolis, Greece; ^16^ Human Genetics Group, Human Cancer Genetics Programme, Spanish National Cancer Research Centre, CNIO, Madrid, Spain; ^17^ Bilkent University, Faculty of Science, Department of Molecular Biology and Genetics, Ankara, Turkey; ^18^ Lebanese American University, Chouran, Beirut, Lebanon; ^19^ Kuban Medical University, Krasnodar, Russia; ^20^ Department of Human and Medical Genetics, Vilnius University, Faculty of Medicine, Vilnius, Lithuania; ^21^ Department of Genetics and Cytology, Karazin Kharkiv National University, Kharkiv, Ukraine; ^22^ Mongolian Academy of Medical Sciences, Ulaanbaatar, Mongolia; ^23^ N.N. Blokhin Russian Cancer Research Center RAMS, Russia; ^24^ Kemerovo State University, Kemerovo, Russia; ^25^ Institute for Humanities Research of the Republic of Bashkortostan; ^26^ N.N. Alexandrov Research Institute of Oncology and Medical Radiology, Minsk, Belarus; ^27^ Department of Radiation Oncology, Hannover Medical School, Hannover, Germany; ^28^ Department of Cartography, Lomonosov Moscow State University, Moscow, Russia; ^29^ Cancer Genome Project, Wellcome Trust Sanger Institute, Wellcome Trust Genome Campus, Hinxton, Cambridgeshire, CB10 1SA, UK; ^30^ University of Edinburgh, Institute of Evolutionary Biology, Edinburgh, Midlothian, Scotland; ^31^ Department of Obstetrics and Gynecology, Hannover Medical School, Hannover, Germany; ^32^ Vavilov Institute of General Genetics RAS, Moscow, Russia

**Keywords:** MDM2, SNP285, SNP309, polymorphism, promoter

## Abstract

The *MDM2* promoter SNP285C is located on the SNP309G allele. While SNP309G enhances Sp1 transcription factor binding and *MDM2* transcription, SNP285C antagonizes Sp1 binding and reduces the risk of breast-, ovary- and endometrial cancer. Assessing SNP285 and 309 genotypes across 25 different ethnic populations (>10.000 individuals), the incidence of SNP285C was 6-8% across European populations except for Finns (1.2%) and Saami (0.3%). The incidence decreased towards the Middle-East and Eastern Russia, and SNP285C was absent among Han Chinese, Mongolians and African Americans. Interhaplotype variation analyses estimated SNP285C to have originated about 14,700 years ago (95% CI: 8,300 – 33,300). Both this estimate and the geographical distribution suggest SNP285C to have arisen after the separation between Caucasians and modern day East Asians (17,000 - 40,000 years ago). We observed a strong inverse correlation (*r* = -0.805; p < 0.001) between the percentage of SNP309G alleles harboring SNP285C and the MAF for SNP309G itself across different populations suggesting selection and environmental adaptation with respect to MDM2 expression in recent human evolution. In conclusion, we found SNP285C to be a pan-Caucasian variant. Ethnic variation regarding distribution of SNP285C needs to be taken into account when assessing the impact of *MDM2* SNPs on cancer risk.

## INTRODUCTION

The protein encoded by the *MDM2* (Mouse Double Minute 2 homolog) gene plays a key role in cell cycle control as a regulator of p53 activity (the protein coded for by the *TP53* gene). It also interacts with several other major proteins involved in cell cycle control and growth arrest, such as pRb and E2F1 [1, 2]. The importance of MDM2 function is illustrated by the fact that *Mdm2* knock-out leads to early embryonic death in mice, which can be reversed by concomitant knockout of the *TP53* gene [3, 4]. Although lacking in some well characterized model organisms, like *D.melanogaster* and *C.elegans*, homologues of *MDM2* have been found in many vertebrates as well as invertebrate species [5]. In humans, *MDM2* is amplified and / or overexpressed in many cancer types [6]. High levels of MDM2 lead to increased inhibition and degradation of p53; thus, MDM2 overexpression has been considered to be one of a set of alternative mechanisms of p53 inactivation in several tumor forms [7].

The polymorphism SNP309T>G (rs2279744), located in the *MDM2* intronic promoter (P2), has been found to be associated with enhanced Sp1 transcription factor binding, thereby leading to increased *MDM2* expression [8]. SNP309G was initially found associated with early cancer onset among individuals carrying *TP53* germline mutations (Li-Fraumeni syndrome) and spontaneous soft tissue sarcomas as well as estrogen receptor (ER) rich breast cancer [8, 9]. Subsequent to the initial discovery of SNP309, potential associations between this variant and cancer risk have been studied across many cancer types and ethnic groups. Overall, the findings indicate that the SNP309G allele is associated with an increased risk of several cancer forms in Asian populations while the results in Caucasians are at variance [10, 11], and a study of >5,000 Western European breast cancer patients [12] yielded no associations with cancer risk. Recently, we reported a second SNP (SNP285 G>C; rs117039649) in the same promoter (P2), only 24 bps upstream of SNP309 [13]. SNP285C is in complete linkage disequilibrium with SNP309G in large previously analyzed Caucasian cohorts (>7,000 individuals; p<1.0 x 10^-10^ [13]). SNP285C reduces the binding strength between the Sp1 transcription factor and the *MDM2* promoter, and is associated with a significantly reduced risk of spontaneous breast, endometrial and ovarian cancer as well as a reduced risk of ovarian cancer among *BRCA1* mutation carriers [13-15]. Further, censoring individuals harboring SNP285C revealed a stronger effect of SNP309 status on the risk of these cancers [13, 15]. In contrast, SNP285C was not associated with an altered risk of prostate cancer [15]. The SNP285C variant was detected in about 8% among healthy individuals in Norway, the Netherlands, and UK, but was absent among Han Chinese, and found at a low frequency (<2%) among Finns [13].

Given that SNP285C seems to be a significant cancer risk reducing factor, any difference related to its frequency across populations will add to our understanding of differences in cancer risk between ethnic groups. Further, this variant may confound cancer risk evaluations related to other SNPs in the *MDM2* gene (such as SNP309) depending on the ethnic population investigated. Based on our previous findings [16], we hypothesized the SNP285C variant to occur among North-Western Europeans only. Here, we determined SNP285C distribution across multiple ethnic groups and estimated its time of origin. Our results are consistent with SNP285C being a pan-Caucasian variant originated shortly after divergence between modern day Caucasians and East Asians, suggesting SNP285C to be a cancer risk modulating factor in all populations of Caucasian origin.

## RESULTS

### Ethnic distribution of MDM2 SNP285C

**Figure 1:**
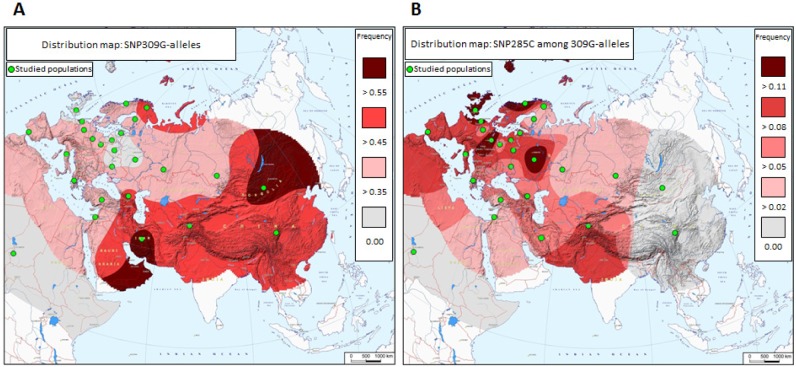
Distribution of *MDM2* promoter SNPs across Eurasia (A) Distribution of SNP309G. Green dots represent populations genotyped. (B) Distribution of SNP285C among SNP309G-alleles.

The genotypes and allele frequencies for *MDM2* SNP285 are summarized in Table 1A/B and Figure 1. The corresponding data for SNP309 is summarized in Supplemental Table S1. For all populations analyzed, the genotype frequencies are in Hardy-Weinberg equilibrium (all p-values >0.3).

In a previous publication, we reported the SNP285C variant to be present in about 7.8 % of North-Western Caucasians (Norway, the Netherlands and UK) but to occur at a low incidence among Finns (1.7%), and to be absent in a cohort of Han Chinese [13]. We therefore hypothesized that SNP285C could be a young variant, present in the North Western part of Europe only. In the present study, we found similar SNP285C frequencies across Western as well as Eastern European populations (with the exception of the Saami and Finnish populations), only dropping off towards the Middle-East (Greece 5.4%, Turkey 4.5%, Lebanon 3.8% and Iran 4.7%) and towards the Eastern Russian regions (Altaians in South Siberia 3.6% and Bashkirs in Ural 3.5%). These data clearly indicate SNP285C to be a pan-European variant (Table 1, Figure 1).

We found no individuals with SNP285C in a sample of ethnic Mongolians (n=229). Together with our previous data from a Chinese cohort, this confirms SNP285C to be absent from East Asian populations. Nor did we find any individuals with SNP285C in a cohort of African-Americans (n=50), strongly indicating this variant to be restricted to Caucasian populations. Notably, the frequency of SNP285C in our new independent cohort of Finns analyzed here (0%; 0 out of 69 individuals), confirmed the low frequency found in our previously reported cohort of Finns (1.7%; 1.2%, the two cohorts taken together; Table 1). As for individuals of Saami heritage, SNP285C was detected heterozygously in one out of 349 individuals only (0.3%; Table 1).

**Table 1A:** Distribution of MDM2 SNP285 genotypes across populations

Population	SNP285 Genotype n (%)			
	GG	GC	CC	Total
Norway*	2274 (92.3)	183 (7.4)	8 (0.3)	2465
Netherlands*	1089 (92.2)	91 (7.7)	1 (0.1)	1181
UK*	320 (92.8)	25 (7.3)	0 (0.0)	345
Germany	264 (92.0)	23 (8.0)	0 (0.0)	287
Italy	277 (92.3)	23 (7.7)	0 (0.0)	300
Poland	424 (91.2)	40 (8.6)	1 (0.2)	465
Belarus	337 (92.6)	27 (7.4)	0 (0.0)	364
Finland (a)*	179 (98.4)	3 (1.7)	0 (0.0)	182
Finland (b)	69 (100.0)	0 (0.0)	0 (0.0)	69
Saami	348 (99.7)	1 (0.3)	0 (0.0)	349
Greece	282 (94.6)	16 (5.4)	0 (0.0)	298
Spain	272 (94.8)	13 (4.5)	2 (0.7)	287
Estonia	285 (94.1)	18 (5.9)	0 (0.0)	303
Lithuania	283 (94.0)	18 (6.0)	0 (0.0)	301
Ukraine	307 (95.9)	12 (3.8)	1 (0.3)	320
Turkey	401 (95.5)	19 (4.5)	0 (0.0)	420
Lebanon	303 (96.2)	12 (3.8)	0 (0.0)	315
Iran	285 (95.7)	13 (4.4)	1 (0.3)	299
Tver/Ryazan (RUS)	155 (91.7)	14 (8.3)	0 (0.0)	169
North Caucasus (RUS)	279 (93.6)	18 (6.0)	1 (0.3)	298
Bashkirs (RUS)	250 (96.5)	9 (3.5)	0 (0.0)	259
Tadjikistan	281 (92.7)	22 (7.3)	0 (0.0)	303
Altaians (RUS)	243 (96.4)	9 (3.6)	0 (0.0)	252
Mongolia	229 (100.0)	0 (0.0)	0 (0.0)	229
China*	319 (100.0)	0 (0.0)	0 (0.0)	319
Afro-Americans	50 (100.0)	0 (0.0)	0 (0.0)	50

**Table 1B:** Distribution of MDM2 SNP285 alleles across populations

Population	SNP285 Alleles n (%)			MAF	285-MAF among 309G-alleles
	G	C	Total		
Norway*	4731 (96.0)	199 (4.0)	4930	0.040	0.118
Netherlands*	2269 (96.1)	93 (3.9)	2362	0.039	0.117
UK*	665 (96.4)	25 (3.6)	690	0.036	0.105
Germany	551 (96.0)	23 (4.0)	574	0.040	0.066
Italy	577 (96.2)	23 (3.8)	600	0.038	0.107
Poland	888 (95.5)	42 (4.5)	930	0.045	0.073
Belarus	701 (96.3)	27 (3.7)	728	0.037	0.111
Finland (a)*	361 (99.2)	3 (0.8)	364	0.008	0.019
Finland (b)	138 (100.0)	0 (0.0)	138	0.000	0.000
Saami	697 (99.9)	1 (0.1)	698	0.001	0.003
Greece	580 (97.3)	16 (2.7)	596	0.027	0.068
Spain	557 (97.0)	17 (3.0)	574	0.030	0.081
Estonia	588 (97.0)	18 (3.0)	606	0.030	0.090
Lithuania	584 (97.0)	18 (3.0)	602	0.030	0.095
Ukraine	626 (97.8)	14 (2.2)	640	0.022	0.063
Turkey	821 (97.7)	19 (2.3)	840	0.023	0.052
Lebanon	618 (98.1)	12 (1.9)	630	0.019	0.043
Iran	583 (97.5)	15 (2.5)	598	0.025	0.046
Tver/Ryazan (RUS)	324 (95.9)	14 (4.1)	338	0.041	0.120
North Caucasus (RUS)	576 (96.6)	20 (3.4)	596	0.034	0.074
Bashkirs (RUS)	509 (98.3)	9 (1.7)	518	0.017	0.042
Tadjikistan	584 (96.4)	22 (3.6)	606	0.036	0.078
Altaians (RUS)	495 (98.2)	9 (1.8)	504	0.018	0.040
Mongolia	458 (100.0)	0 (0.0)	458	0.000	0.000
China*	638 (100.0)	0 (0.0)	638	0.000	0.000
Afro-Americans	100 (100.0)	0 (0.0)	100	0.000	0.000

* Previously published data (Knappskog et al 2011).

### Validation data set for population distribution of MDM2 SNP285C

In order to validate our findings with respect to the variant frequencies, we mined the 1000 Genomes project data bank (www.1000genomes.org), where *MDM2* SNP309 and SNP285 status are available for most individuals. Even though the number of individuals here is limited with respect to each ethnic group, these additional data support our findings: the SNP285C was absent in the Asian populations (Chinese, n=197, Japanese, n = 89), while the frequencies (4.1 – 7.1%) in the Caucasian populations (Americans, British, Spanish and Italians) were in line with our observations. Notably, our finding of a low incidence of SNP285C among Finns was also validated in the 1000 Genomes data-set (1%). Regarding African populations, two individuals (1 out of 97 Nigerians and 1 out of 88 Kenyans) were registered with a SNP285GC genotype in the 1000 Genome project databank. However, taking into account that the quality scores for the SNP-calling of SNP285 is rather poor for these two samples, and the fact that both individuals harbor the SNP309TT genotype, the chance exists that the registered C-alleles may be artefacts.

### Distribution of MDM2 SNP285C among SNP309G-alleles

Since the SNP285C variant is present only in haplotypes with the SNP309G allele (100% linkage disequilibrium with SNP309G), we tested whether any of the differences in SNP285C frequencies between populations could be “passenger-effects” of the observed differences in SNP309 distribution (Supplemental Table S1). In order to remove the potential confounding effects of different SNP309G frequencies, we compared the ratios of SNP285C-alleles divided by the total number of SNP309G-alleles between the individual populations. Table 1B gives the minor allele frequencies (MAFs) for SNP285C among SNP309G alleles. In general, we found the same patterns as for the genotypes described above: the frequency of the SNP285C / 309G haplotype is quite consistent across most Western and Eastern European populations (0.066 - 0.120) but low among Saami and Finns (0.003 and 0.013, respectively) and dropping off towards East Russia and Central Asia, while being absent in East Asia and among individuals of African American heritage.

### Inverse correlation between SNP285 and SNP309 MAFs

**Figure 2:**
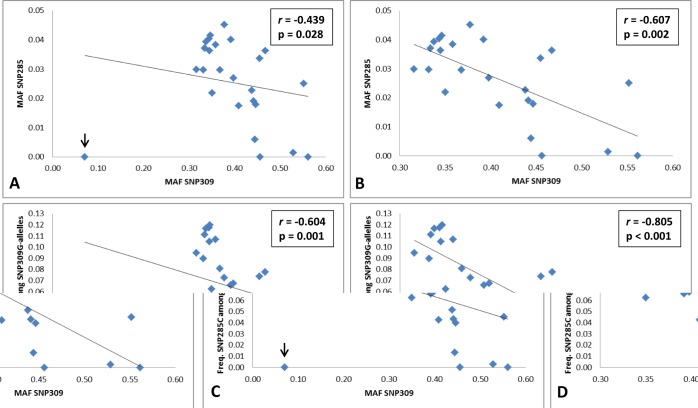
Inverse correlation between *MDM2* SNP285 (rs117039649) and SNP309 (rs2279744) (A-B) Correlation plots for MAF SNP285 and MAF SNP309 including all 25 analyzed populations (A) and 24, excluding the special case of African Americans where SNP285 is absent and the frequency of SNP309G is very low (B). (C-D) Correlation plots for the frequency of SNP285C among SNP309G-alleles in all 25 analyzed populations (C) and excluding African Americans (D).

When comparing the frequencies of SNP285C and SNP309G across the populations (Table 1 and Supplemental Table S1), surprisingly, we found an *inverse* correlation (r = -0.439, p = 0.028; Figure 2A). This correlation remains after removing the special case of African Americans, where SNP285C is absent and the frequency of SNP309G is very low (r = -0.607, p = 0.002; Figure 2B), as well as after removing all populations where SNP285C is absent (African Americans, Chinese and Mongolians) from the calculations (r = -0.519, p = 0.013; correlation plot not shown).

Taking into account the fact that the SNP285C variant is found on the SNP309G haplotype only, we calculated the correlation between the incidence of the SNP285C/309G haplotype among SNP309G alleles and the respective SNP309 MAF across the different populations. This strengthened the inverse correlation (r = -0.604, p = 0.001 and r = -0.805, p < 0.001, including and excluding African Americans, respectively; Figure 2C-D).

### Ancestry of MDM2 SNP285C

To obtain an estimate of the age of SNP285C, we used short tandem repeats (STR) variants surrounding the *MDM2* locus. Screening 14 STRs in 48 healthy Norwegian males, we identified two STRs (D12S1680 and D12S1693) generating a haplotype (CA)_21_-(CA)_13_ in statistically significant LD with SNP285C. Based on this haplotype, the spreading time (age) of SNP285C was estimated to be 586 generations (95% CI: 333 – 1333). Applying an average generation time of 25 years, we estimated SNP285C to be approximately 14,700 years old (95% CI: 8,300 – 33,300). Interestingly, recent estimates have indicated the separation of present day Caucasians and East Asians to have occurred around 23,000 years ago (95% CI: 17,000 – 43,000) [17]. Thus, our STR analysis, consistent with the geographical distribution of SNP285C, suggests this variant to have arisen after the separation of Caucasians from modern day East Asians.

### Haplotype diversity for MDM2 SNP285C versus SNP285G

**Figure 3:**
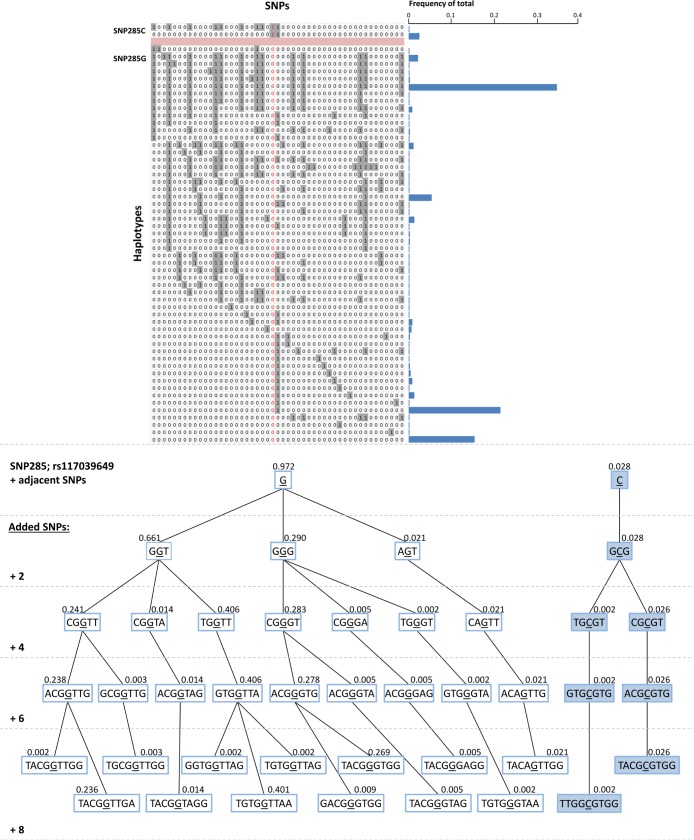
(A) Imputed haplotypes including 49 SNPs within / flanking the MDM2 gene, extracted from 288 Caucasian individuals; data extracted from the 1000 genome project - www.1000genomes.org. SNP285 (rs117039649) is indicated in red. “1” indicates presence of the minor allele for each SNP, while “0” indicates the presence of the major allele. Frequency of the different unique haplotypes (n=56) observed among the 576 alleles (288 individuals) are indicated as blue horizontal bars. (B) Haplotype-tree based on 8 SNPs surrounding SNP285 (rs117039649). The tree and the frequencies given for each haplotype are based on the same 288 individuals as in Figure 3A.

Finally, we mined the data from the 1000 Genomes project, assessing potential differences with respect to haplotype diversity between the SNP285C and G alleles. While STR genotypes are not available, we extracted genotypes for 49 SNPs (including SNP285 and 24 SNPs on each side; Supplemental Table S2) in 288 Caucasian individuals, enabling us to compare the diversity between the alleles carrying SNP285G with those harboring the C-variant, across a region of approximately 8.7 kilobases (chr12:69197737 – 69206479). The imputed haplotypes are depicted in Figure 3A. Among the many haplotypes (n=54) carrying the SNP285G-allele, three were dominant with respect to frequency while a high number of haplotypes were observed in low frequencies (Figure 3A). In the haplotypes carrying SNP285C (n=16), we observed 15 identical ones and a single haplotype carrying a SNP-pattern similar to several of the haplotypes with SNP285G in the region upstream of this SNP, indicating a recombination to have taken place just upstream of SNP285.

Regarding SNP285 as the focal point and assessing the haplotype branching based on the eight nearest neighboring SNPs, we observed a much more complex branching pattern for the SNP285G-haplotypes than for SNP285C (Figure 3B). These haplotype data indicates SNP285C to be much younger than SNP285G, consistent with a selective sweep of this variant.

## DISCUSSION

While multiple SNPs have been detected throughout the *MDM2* promoters, effects on transcription and pathophysiological effects, such as associations with cancer risk, have been described for a few only. While SNP309 (rs2279744) seems to have an oncogenic effect, and SNP285C (rs117039649) is associated with a reduced risk of certain cancers, we found a third SNP in the same region (SNP344; rs1196333) not to be associated with altered risk of any of several major tumour forms [18].

So far, apart from our own studies [13, 15], only a few studies have assessed SNP285 status. Paulin and co-workers found SNP285C in 4.4% of healthy Scottish individuals [19], while Piotrowski and colleagues found the variant C-allele in 10.2 % of healthy Polish individuals [20]. Further, Ryan and colleagues found 6% of Americans of Caucasian ancestry to harbor SNP285C [21], while Renaux-Petel and co-workers found this variant among 5.2% of French individuals diagnosed with germline *TP53* mutations [Li-Fraumeni patients; 22]. Although Ryan et al detected SNP285C in 1% of African Americans (3 out of 253 genotyped individuals), this observation does not contradict the lack of SNP285C in Africans populations, as it has been estimated that on average 20% of the gene pool in the modern day African-American population is of European origin [23]. Taken together, these data are all in line with our present findings.

The conclusions from the data presented here are also supported by the SNP status from several ethnic groups through the genomic data available from the 1000 Genome project. Further, the distribution pattern observed for SNP285C has many parallels known for other human genetic variants, including mitochondrial DNA and Y-chromosomal haplogroups, being so-called West Eurasian [24-26].

The reason for the low frequency of SNP285C among individuals of Finnish and Saami heritage compared to other European populations is unknown. However, the Finnish population is known to have undergone narrow bottlenecks, and its genetic composition differs from other European populations [27]. Notably, the low frequency of SNP285C among Finns was detected in two independent cohorts and further confirmed in the 1000 Genome project. Regarding the Saami population, it is generally assumed to have originated from a narrow subset of Europeans, although their entry route to Northern Scandinavia remains an issue of debate [28].

Based on the STR analysis, we estimated the age of SNP285C to be about 14.700 years. Calculations based on nuclear DNA divergence has estimated the separation between the European and Asian population to have occurred around 23,000 (CI: 17,000 - 40,000) years ago, [17], while calculations based on mitochondrial DNA divergence have estimated this event to take place approximately 40,000 years ago [29]. Thus, our age estimate, similar to our findings with respect to geographical distribution of SNP285C, indicates that this variant has arisen early after separation of the two population groups.

It should be noted, however, that several theories regarding human expansion out of Africa and into the rest of the world exist. Probably, neither the expansion of humans from Africa nor the split between Caucasians and Asians have occurred as single events [30] and our model may therefore be too simplistic. Although less likely, based on our age estimations and the haplotype diversities, SNP285C may have originated early and existed in other ethnic groups, including Asians and Africans, only to subsequently disappear during evolution.

Interestingly, we detected a strong inverse correlation between the frequency of the SNP285C/309G haplotype and SNP309G MAF across the different populations analyzed. While it is not possible to draw a definite conclusion with respect to the causes of this inverse correlation, it seems clear that this finding may not be explained by simple bottleneck effects and subsequent rapid expansion of evolutionary neutral variants in a population. The fact that SNP285C and SNP309G have opposite effects on *MDM2* transcription [13] raise the question whether such an inverse correlation may be due to environmental adaption across different geographic areas. Notably, applying a mathematical formulation for dating of evolutionary neutral polymorphisms (generations = -4N_e_ [p(ln_e_ p) + (1 – p)(ln_e_(1-p)]) [31, 32], with a haplotype frequency of 3-4%, SNP285C may be estimated to have arisen around 85,000 years ago. The CI for such an estimate however will be large [33] precluding any firm conclusions to be drawn, but the discrepancy between this estimate, the data from geographical distribution and STR-based analyses adds support to the hypothesis that SNP285C is not evolutionary neutral.

While different studies have shown SNP309 and SNP285 to affect the risk for different cancer forms [10, 11], any difference with respect to cancer risk is unlikely to have played a major role in evolution, as most cancers occur after reproductive age. MDM2 is a key factor in multiple cellular processes; thus, it is likely that fine-tuning of its activity in response to environmental influences is important. Notably, SNP309 status has been associated with missed abortions [34] and with male [35] and female [36] infertility. Taken together, our findings indicate both SNP285 and SNP309 status may affect biological processes of evolutionary importance. SNP285 and SNP309 have been shown to reduce and enhance transcription-factor Sp1 binding to the *MDM2* promoter, respectively [13]. Further, they have been shown to have opposing effects on cancer risk. Thus, it is not unreasonable to postulate they may have antagonistic effects on other biological mechanisms as well.

In conclusion, detailed mapping of the prevalence of SNP285C in multiple populations indicates this variant to be spread through most populations of Caucasian origin. The status of this cancer risk reducing SNP needs to be taken into account when assessing the impact of other *MDM2* SNPs on cancer risk across different ethnic and geographic populations.

## MATERIALS AND METHODS

### Subject cohorts

In the present project, we screened SNP285 and SNP309 genotypes in 5,937 healthy individuals, representing 21 different populations / cohorts. The populations and the number of individuals included in each of them are listed in Table 1. In addition, we included data from our previous study [13] making the total number healthy individuals genotyped 10,429, representing 25 different populations / cohorts (Table 1), thereby allowing us to determine the geographic distributions of both variants.

In order to validate our findings, we performed data mining and extracted information regarding *MDM2* SNP285 and SNP309 status from genomic information from the 1000 Genomes project (www.1000genomes.org). Here, we retrieved SNP status from multiple ethnic groups mounting up to a total of 1,094 further individuals.

### SNP genotyping

The Finnish, Estonian, Lebanese, German and Chinese samples were genotyped for SNPs 285 and 309 in their respective countries by PCR amplification and Sanger sequencing (the three former populations) or restriction fragment length analysis (the two latter populations), as previously described [13, 37-39]. Norwegian, Dutch and British samples were previously genotyped in Norway by PCR amplification and Sanger sequencing [13].

The remaining samples were analyzed, for both *MDM2* SNP285 and 309 status using the LightSNiP technology (TIB-MOLBIOL) on a LightCycler 480 instrument (Roche) in our laboratory in Bergen, Norway. These amplifications were performed in a final reaction volume of 10 μl, containing 1 μl LightCycler® FastStart DNA Master HybProbe mix (Roche Diagnostics), 0.5 μl LightSNiP mix (TIB MOLBIOL), 3 mM MgCl_2_ and 10 - 50 ng DNA. The thermocycling and melt curve conditions were set according to the optimized protocol provided by TIB MOLBIOL: 10 minutes initial denaturation / activation at 95 °C, followed by 45 cycles of denaturation at 95 °C for 10 seconds, annealing for 10 seconds at 60 °C and elongation at 72 °C for 15 seconds. Subsequently, the high resolution melting (HRM) step was performed, starting with an initial denaturation at 95 °C for 30 seconds, followed by melting from 40 °C to 75 °C with a ramp rate of 0.19 °C/sec and a final cooling step at 40 °C for 30 seconds. The HRM curve profiles were then analyzed using the Melt Curve Genotyping module in the LightCycler® 480 software version 1.5. Both Light SNiP assays (for SNP285 and SNP309) had identical thermocycling conditions.

For assay quality control, 3 % of the samples analyzed by LightSNiP technology had their genotypes validated by Sanger sequencing. The concordance between the two methods was 100%, assuring no bias in the data sets based on method of genotyping.

### Age estimation of SNP285 by STR genotyping

14 STRs surrounding the *MDM2* promoter P2 were genotyped by PCR amplification and size analyses of the amplified products. The STRs and the primers used for amplification are listed in Supplemental Table S3. Amplification was performed in a 50 μl reaction mix containing 0,5 μl Dynazyme EXT polymerase (Finnzymes), 1x Dynazyme reaction buffer, 5% DMSO, 0.2 mM dNTPs, 0.2 μM of each primer and 10-50 ng DNA. The thermocycling conditions were a 5 minutes initial denaturation / activation step at 95 °C, followed by 40 cycles of 45 seconds denaturation at 95 °C, 45 seconds annealing at 55 °C and 45 seconds elongation at 72 °C. The amplified products were separated by capillary electrophoresis on a ABI3700 sequencer. The STR genotyping was performed in a selection of 48 healthy Norwegian individuals with known status for *MDM2* SNPs 285, 309 and 344.

Haplotypes were imputed from the SNP and STR genotype data, using the Arlequin software v3.5 [40].

The only haplotype (CA)_21_-(CA)_13_ at two STRs D12S1680 and D12S1693 for which LD with SNP285C was statistically significant was identified and used for an estimation of the age of SNP285C. We applied the formula g = log δ / log (1 - θ) [41], where g equals the time (in generations) back to the initial spreading of mutant chromosomes in population, δ equals the measure of LD and θ is the recombination fraction between the disease locus and the marker. In this case, “mutant” chromosomes are SNP285C bearing chromosomes, the “disease locus” is the *MDM2* gene and the “marker” is the two STRs with LD haplotype. To assess the degree of LD, we applied the formula δ = (P_D_ – P_N_) / (1 – P_N_) [42], where P_D_ is the frequency of the associated alleles on the diseased chromosome (bearing SNP285C in this case) and P_N_ is the frequency of the same allele on the control chromosome (not bearing SNP285C). D12S1680 and D12S1693 are located in the same linked cluster on genetic Marshfield map and so the haplotype (CA)_21_-(CA)_13_ was used as a single associated allele in the formula. CI for δ was calculated as previously described [43].

### Haplotype diversity

Haplotype diversity for the SNP285G and C-alleles were assessed by extracting SNP information from the 1000 Genomes dataset (www.1000genomes.org). We extracted SNP-data from 288 individuals, including all Caucasian individuals (possible carriers of SNP285C) except for Finns (where the frequency of SNP285C was expected to be very low). From these individuals, the genotypes of 49 SNPs (Supplemental Table S2), including SNP285 and its 24 flanking SNPs on each side (spanning a region of 8.7 kb; chr12:69197737 - 69206479) were phased using Impute2 [44].

### Statistics

Comparisons of genotype distributions between population groups were performed by Fisher's exact tests (2x2 comparisons) and Chi-square tests (2x*n* comparisons), using SPSS software, version 18. All p-values given are two-sided, and p-values obtained by the Fisher's exact tests are given as cumulative.

## SUPPLEMENTARY INFORMATION AND TABLES


