# Supplementary material for: Population distribution and ancestry of the cancer protective MDM2 SNP285 (rs117039649)
Source: Oncotarget. 2014 Apr 18;5(18):8223–34. doi: 10.18632/oncotarget.1910 (PMC4226679; doi:10.18632/oncotarget.1910)
Supplement: Supplementary file 1 [file oncotarget-05-8223-s001.pdf]

Population distribution and ancestry of the cancer protective MDM2 SNP285 (rs117039649)

Supplementary Information

Supplemental Table S1: Distribution of *MDM2* SNP309 genotypes and alleles across populations.

Population	SNP309 Genotype *n* (%)				MAF
	TT	TG	GG	Total	
Norway*	1072 **(43.5)**	1093 **(44.3)**	300 **(12.2)**	2465	0.343
Netherlands*	529 **(44.8)**	507 **(42.9)**	145 **(12.3)**	1181	0.337
UK*	142 **(41.2)**	168 **(48.7)**	35 **(10.1)**	345	0.345
Germany	108 **(37.6)**	133 **(46.3)**	46 **(16.0)**	287	0.392
Italy	114 **(43.5)**	157 **(52.3)**	29 **(9.7)**	300	0.358
Poland	177 **(38.0)**	225 **(48.4)**	63 **(13.5)**	465	0.377
Belarus	168 **(46.2)**	149 **(40.9)**	47 **(12.9)**	364	0.334
Finland (a)*	54 **(29.7)**	97 **(53.3)**	31 **(17.0)**	182	0.437
Finland (b)	19 **(27.5)**	36 **(52.2)**	14 **(20.3)**	69	0,464
Saami	76 **(21.8)**	177 **(50.7)**	96 **(27.5)**	349	0.529
Greece	94 **(31.5)**	171 **(57.4)**	33 **(11.1)**	298	0.398
Spain	109 **(38.0)**	145 **(50.5)**	33 **(11.5)**	287	0.368
Estonia	124 **(40.9)**	157 **(51.8)**	22 **(7.3)**	303	0.332
Lithuania	143 **(47.5)**	126 **(41.9)**	32 **(10.6)**	301	0.316
Ukraine	132 **(41.3)**	152 **(47.5)**	36 **(11.3)**	320	0.350
Turkey	142 **(33.8)**	188 **(44.8)**	90 **(21.4)**	420	0.438
Lebanon	104 **(33.0)**	144 **(45.7)**	67 **(21.3)**	315	0.441
Iran	60 **(20.1)**	148 **(49.5)**	91 **(30.4)**	299	0.552
Tver/Ryazan (RUS)	70 **(41.4)**	81 **(47.9)**	18 **(10.7)**	169	0.346
North Caucasus (RUS)	81 **(27.2)**	163 **(54.7)**	54 **(18.1)**	298	0.455
Bashkirs (RUS)	92 **(35.5)**	122 **(47.1)**	45 **(17.4)**	259	0.409
Tadjikistan	89 **(29.4)**	145 **(47.9)**	69 **(22.8)**	303	0.467
Altaians (RUS)	73 **(29.0)**	133 **(52.8)**	46 **(18.3)**	252	0.446
Mongolia	42 **(18.3)**	117 **(51.1)**	70 **(30.6)**	229	0.561
China*	94 **(29.5)**	159 **(49.8)**	66 **(20.7)**	319	0.456
Afro-Americans	44 **(88.0)**	5 **(10.0)**	1 **(2.0)**	50	0.070

* Previously published data (Knappskog et al 2011).

Supplemental Table S2: SNPs on chromosome 12 included in haplotype diversity analysis.

SNP#	rs ID	POS Chr12	REF	ALT
SNP1	rs2088577	69197737	A	C
SNP2	NA	69197863	G	A
SNP3	rs112886224	69198025	C	G
SNP4	rs2088578	69198112	G	A
SNP5	rs79825043	69198294	T	C
SNP6	rs1144941	69198631	C	T
SNP7	NA	69198763	G	A
SNP8	rs2120742	69199207	C	T
SNP9	NA	69199356	A	T
SNP10	rs1144942	69199362	A	C
SNP11	NA	69199478	C	T
SNP12	NA	69199525	T	C
SNP13	rs1144943	69199745	T	G
SNP14	rs1144944	69200485	A	G
SNP15	rs3730486	69201242	C	T
SNP16	NA	69201394	T	A
SNP17	rs1144945	69201598	A	T
SNP18	rs937282	69201797	C	G
SNP19	NA	69201962	C	T
SNP20	rs3730492	69202015	T	G
SNP21	rs937283	69202164	A	G
SNP22	rs2870820	69202326	C	T
SNP23	NA	69202378	G	A
SNP24 («SNP285»)	rs117039649	69202556	G	C
SNP25 («SNP309»)	rs2279744	69202580	T	G
SNP26 («SNP344»)	rs1196333	69202615	T	A
SNP27	NA	69202912	G	A
SNP28	rs3730495	69203312	G	A
SNP29	NA	69203496	A	T
SNP30	rs1196335	69203524	T	A
SNP31	rs3730498	69203554	T	G
SNP32	rs114128261	69204248	A	T
SNP33	NA	69204484	G	A
SNP34	NA	69204706	C	T
SNP35	rs3730499	69204837	G	A
SNP36	rs3730500	69204871	C	T
SNP37	rs55956268	69204883	G	C
SNP38	rs3730502	69205046	C	G
SNP39	rs3730503	69205202	T	C
SNP40	rs3730504	69205287	A	G
SNP41	rs1690924	69205321	T	C
SNP42	rs1196337	69205660	A	G
SNP43	rs3730507	69205675	T	C
SNP44	rs3730508	69205723	A	G
SNP45	rs1196338	69205751	C	T
SNP46	NA	69205919	T	C
SNP47	rs3730510	69205953	G	A
SNP48	NA	69206128	G	A
SNP49	rs1663577	69206479	A	G

Supplemental Table S3: Primers used for genotyping of STRs surrounding the *MDM2* promoter.

STR	Coordinates	Left primer	Right primer	Fluorochrome	Size Range
D12S1700	12:60016773-0016952	AGATGCTAATGCCATGACA	CCCTGTGGATGACCAGTT	FAM	169-183
D12S1662	12:60844531-0844726	TGCAGAACTGTGAGAAACAA	TCCTGAAATATGTAGTGTGAAATG	CY3	166-198
D12S83	12:60889388-0889472	TTTTTGGAAGTCTATCAATTTGA	TAGCAGAGAAAGCCAATTCA	CY3	085-099
D12S1726	12:62459870-2460044	TGCTTGAGGTGATGGATAC	TAAAAGGCTGGATAACACTG	FAM	175-181
D12S1610	12:64974315-4974455	TCCTGGGGGATAGAATGAG	GACACCTTGATTTGGGCTT	JOE	119-149
D12S1686	12:65665173-665403	CTAGGGTCAGAGTTCCTGCT	CACTGTCCAATCAAGTAAGGC	CY3	187-251
D12S1702	12:67359257-7359516	AGATGGGTAAAGGGCA	AGGTATCTATGAGGGGGTT	JOE	216-272
D12S1601	12:67540842-7541036	AGCTACTTCAGAAAACTATTTGACA	GCAGGAACTTAACTCTTGCTT	JOE	193-203
D12S1680	12:69915944-9916203	AAAAATACCATCACAGACTACTACG	ATCAAGGATATTGGCTGAAG	FAM	244-266
D12S1693	12:70226709-0226796	CGTAAAATGTCCCAAATGAA	AGCTGAGGAACAGGCTTG	FAM	070-088
D12S1722	12:71012784-1013026	AAGGAACTTGCCCAAGATTA	GGGAATACTCTGATCCCCA	JOE	235-249
D12S1711	12:71716499-1716687	GTGCCCTGATGTTGGT	GGCAAAATAGACCTGAAGTC	CY3	173-227
D12S80	12:72023241-2023451	CCAGCCTGGAATGATATGTA	GAATGTCAATGGACCAGATG	CY3	207-223
D12S1660	12:76462424-6462624	GTTTGACACAAGTACACCAAGTCAC	AAGTACCAGCCTATTTTTTTGGACC	FAM	196-226
